# Systematic analysis of secondary life cycle inventories when modelling agricultural production: A case study for arable crops

**DOI:** 10.1016/j.jclepro.2017.03.179

**Published:** 2018-01-20

**Authors:** Sara Corrado, Valentina Castellani, Luca Zampori, Serenella Sala

**Affiliations:** a Università Cattolica del Sacro Cuore, Istituto di Chimica Agraria e Ambientale, Via Emilia Parmense 84, 29122 Piacenza, PC, Italy; b European Commission, Joint Research Centre, Directorate D - Sustainable Resources - Bio-Economy Unit, Via Enrico Fermi 2749 TP290, I-21027 Ispra, Italy

**Keywords:** Life Cycle Assessment, Life cycle inventory, Agriculture, Arable crops, Databases

## Abstract

Analysis of agricultural production with life cycle based methodologies is data demanding. To build comprehensive life cycle inventories, secondary datasets are commonly used when primary data are not available. However, different inventory data and modelling approaches are used to populate secondary datasets, leading to different results. The present study analyses the features of twelve secondary datasets to support datasets selection and proper interpretation of results. We assess twelve datasets for arable crop production in France, as modelled in three databases often used in the LCA field (Agri-footprint, ecoinvent and AGRIBALYSE). First, we compared system boundaries and general assumptions. Second, we focused on foreground systems comparing, inventory data, data sources and modelling approaches. Third, we performed a contribution analysis of impact assessment results to identify modelling choices that contribute most to differences in the results. Nine relevant elements were identified and assessed: definition of system boundaries and modelling of agricultural practices, characteristics of inventory data, agricultural operations, fertiliser application and fate, plant protection products application and fate, heavy metals inputs to the agricultural system and fate, irrigation assumptions, land use and transformation. The datasets differ greatly with respect to these elements. Hence, recommendations are drawn from the datasets comparison, supporting the selection of the datasets coherently with the goal and scope of a study and interpretation of results.

## 1 Introduction

Assessment of environmental profiles of the food supply chain is increasingly needed in the context of sustainable production and consumption initiatives. The aim is to identify drivers of environmental impacts associated with food production and possible improvements thereof. Life Cycle Assessment (LCA) is a reference methodology for supply-chain impact assessment (ISO, 2006). However, when the subject of the study is a manufactured product (e.g. a food product), data on agricultural stages of basic ingredients (e.g. wheat) are often not collected directly, relying instead on “secondary data” (Williams et al., 2009). This approach helps to streamline estimation of the product's environmental profile (Teixeira, 2015), reducing the resources required to collect data and allowing a LCA to be performed when the necessary life cycle inventory data are not available from primary sources of data. The choice of the secondary datasets to be used is considered one of the challenges for a robust LCA study (Notarnicola et al., 2017) and can influence the results of the LCA study (e.g. Peereboom et al., 1998, found out a variation of impact results from 10% to 100% when different datasets were used in a case study on PVC). Indeed, different modelling assumptions in datasets aiming to represent the same product system can lead to different results, affecting the reliability of the LCA study (Williams et al., 2009). LCA practitioners are, therefore, recommended to choose datasets carefully according to the goal and scope of their studies (Fazio et al., 2015).

Several authors have already analysed secondary data from different points of view: (i) developing criteria for assessing data quality (e.g. Garraín et al., 2015, Grabowski et al., 2015), (ii) estimating influence of datasets quality on life cycle impact assessment (LCIA) results (Peereboom et al., 1998), (iii) developing approaches based on a descriptive and statistical analysis to assess reliability of secondary data used in LCA (Teixeira, 2015), and (iv) adopting meta-analysis to estimate average values of environmental impacts (e.g. Achten and Van Acker, 2015).

To our knowledge, however, systematic analysis of secondary datasets modelling arable crops has not been performed to date. Hence, the present study analyses secondary datasets of arable crop production, based on the approach adopted by Peereboom et al. (1998) with some adaptations for the agricultural context. It aims to understand similarities and differences in datasets of arable crop cultivation and the extent to which the differences may affect LCIA results. We identified and analysed elements in datasets which may influence LCA results the most, as well as strengths and weaknesses of the modelling approaches adopted. Results of the present study could help LCA practitioners to choose secondary datasets which are consistent with the goal and scope of their study and interpret results properly. Furthermore, the results may inform dataset developers about the need for potential improvements to, for example, modelling approaches and underlying assumptions on which datasets were built.

The article is organised as follows: first, system boundaries and underlying assumptions adopted in secondary datasets for arable crops within three databases are reviewed. Second, a summary of the approaches adopted to model the foreground system is supplemented by highlighting similarities and differences among the approaches. Next, the influence that modelling approaches can have on LCIA is illustrated. Finally, conclusions about relevant elements of the datasets are provided.

## 2 Materials and methods

The present study is focused on analysis of secondary datasets for arable crop production as modelled in three of the most commonly used LCA databases: AGRIBALYSE^®^ v 1.2 (Colomb et al., 2015), Agri-footprint^®^ v 1.0 (Blonk Agri-footprint BV, 2014a) and ecoinvent^®^ v 3.1 (Weidema et al., 2013). Four arable crops cultivated in France in all three databases were selected for analysis: soft wheat, barley, rapeseed and pea (Table 1). Agro-footprint and ecoinvent do not include specific datasets modelling soft wheat production, therefore the dataset for wheat production was considered in the analysis. Futhermore, as AGRIBALYSE includes both spring pea and winter pea, the average of the two was considered in the analysis. The crops and their country of production were selected to obtain the largest number of comparable datasets. Ecoinvent includes: attributional datasets, consequential datasets and datasets based on the so-called “cut-off system model” approach (whose underlying philosophy is that primary production of materials is always allocated to the primary user of a material) (ecoinvent, 2016). Differences in the LCIA results of these three modelling approaches were screened. As few differences were found (supplementary material, Figs. S6–S9) and inclusion of consequential and “cut-off” datasets would have rendered the comparison with other databases too complex, only ecoinvent's attributional datasets (the default) were analysed.

**Table 1:** Databases assessed in the study., FR = France, U = unit process, S = system process, Alloc def = ecoinvent default allocation.

Database	Dataset
AGRIBALYSE v 1.2	Soft wheat grain, conventional, national average, at farm gate/FR U Barley, conventional, malting quality, national average, at farm gate/FR S Rapeseed, conventional, 9% moisture, national average, at farm gate/FR U Winter pea, conventional, 15% moisture, at farm gate/FR U Spring pea, conventional, 15% moisture, at farm gate/FR U
Agri-footprint v 1.0	Wheat grain, at farm/FR Barley grain, at farm/FR Rapeseed, at farm/FR Pea, at farm/FR
ecoinvent v 3.1	Wheat grain {FR} | wheat production | Alloc Def, U Barley grain {FR}| barley production | Alloc Def, U Rape seed {FR}| production | Alloc Def, U Protein pea {FR}| production | Alloc Def, U

**Fig. 1:**
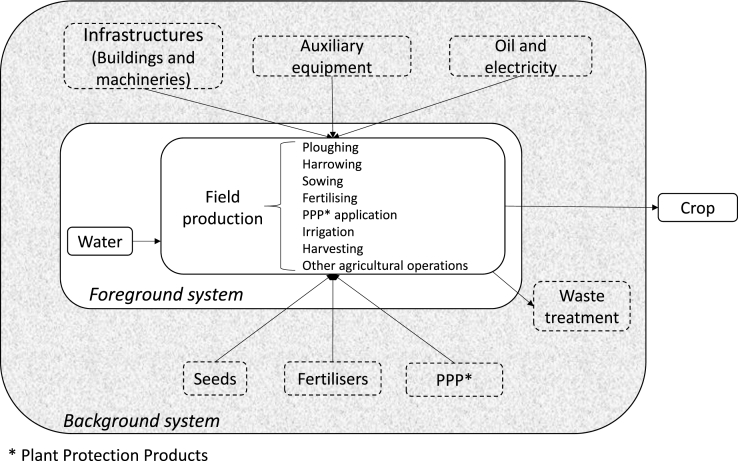
Representation of agricultural production systems. Adapted from Hayashi et al. (2006).

Datasets were analysed based on information reported in dataset documentation, data provided in the databases as implemented in the software SimaPro v 8.0.5, and some other relevant publications (Frischknecht and Rebitzer, 2005, Nemecek et al., 2014). We considered a generic representation of an agricultural production system and distinguished foreground and background systems when analysing datasets (Fig. 1). System boundaries and underlying assumptions of each dataset were compared referring to this diagram. The foreground system was examined by describing the assumptions adopted to model it, highlighting similarities and differences among datasets per hectare of cultivated land. For wheat and barley, all inputs and output flows of cropping were allocated to the grain (none to the straw) to allow results to be compared. As with the foreground system analysis, allocation of potential impacts among co-products was removed from the inventories. Furthermore, since Agri-footprint includes datasets modelling the same product with different allocation approaches, a screening of the effect of allocation on LCIA was performed. LCIA was conducted for the three datasets using the ILCD Midpoint v 1.06 characterisation method (EC-JRC, 2011) as implemented in the software SimaPro v. 8.0.5. We assessed potential impacts of 1 kg of product at the farm gate. We performed three types of analysis:

• comparison of system boundaries and underlying assumptions
• analysis of how the foreground system is modelled, focusing on: agricultural operations, fertiliser application and nutrient fate; plant protection product (PPP) application and fate; heavy metal (HM) input, mass balance and fate; irrigation; land occupation and transformation
• comparison of LCIA results of the foreground system, including the relative contribution of the background system.

When comparing inventory data and LCIA results, uncertainty data provided within the datasets were taken into account, and differences among data were considered statistically significant when 95% confidence intervals did not overlap. The 95% confidence intervals were estimated using Monte-Carlo simulation with 500 replicates.

## 3 Results

One substantial difference among the datasets is the source of activity data from which the inventories were developed. AGRIBALYSE and Agri-footprint data were derived from average information for the French context, collected respectively using questionnaires distributed to technical institutes and from available statistics or other specific data (e.g. the literature). In contrast, ecoinvent datasets were built from data collected for a single French region, Barrois, in the GL-Pro project (Nemecek and Baumgartner, 2006).

Database providers checked the quality of activity data. For AGRIBALYSE and Agri-footprint, the quality check was performed by experts not directly involved in defining the inventory data and quality was analysed at two levels: plausibility of activity data and presence of data gaps or errors in the LCIA and LCA results (Blonk Agri-footprint BV, 2014a, Koch and Salou, 2013). Ecoinvent datasets, instead, were independently reviewed before they were integrated into the ecoinvent database. Data quality was assessed based on the pedigree-matrix approach in AGRIBALYSE and ecoinvent (Frischknecht and Rebitzer, 2005, Weidema et al., 2013), which, by qualitatively assessing data quality indicators, is applicable when only a single mean value for activity data is available (Frischknecht and Jungbluth, 2007). The pedigree matrix considers information about the quality of each primary input and output datum in terms of reliability, completeness, temporal correlation, geographical correlation and further technological correlation.

Uncertainty in activity and inventory data cannot be avoided due to variability and stochastic errors in activity data, appropriateness of input and output flows, model uncertainty and the exclusion of important flows (Frischknecht and Jungbluth, 2007). Therefore, a basic uncertainty is reported in ecoinvent for “unit process” datasets. In AGRIBALYSE, barley production is reported only as a “system process” with no information about uncertainty. In Agri-footprint, uncertainty is estimated only for certain categories of background data, mainly according to expert knowledge (Blonk Agri-footprint BV, 2014a). The lack of information about uncertainty partially influenced analysis of each dataset's foreground.

### 3.1 Analysis of system boundaries and underlying assumptions

**Fig. 2:**
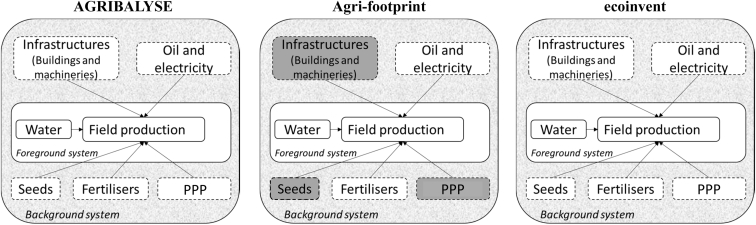
System boundaries of the datasets considered in the study. White boxes represent processes included in inventories, whereas grey ones represent processes not included in inventories.

System boundaries and the main underlying assumptions adopted to model arable crop production differ somewhat among the three databases (Fig. 2, Table 2). It is evident that, within a given database, the same modelling approach is adopted for all the arable crops analysed. Furthermore, databases have several similarities in how they model agricultural systems. In particular, AGRIBALYSE background data were taken from ecoinvent v 2.2, which explains some similarities between the two databases. However, differences among the databases were observed.

**Table 2:** Underlying assumptions and modelling approaches adopted to build the datasets analysed, as described in their documentation.

		AGRIBALYSE	Agri-footprint	ecoinvent
Data source		Provided by technical institutes (e.g. ARVALIS – Institut du Végétal)	Multiple sources (e.g. scientific literature, official statistics such as FAOstat, Eurostat)	GL-Pro project – Barrois region, France (Nemecek and Baumgartner, 2006)
Straw management (when applicable)		Partly removed from the field	Completely removed from the field	Left on the field
Allocation of co-products (grain and straw)		Not performed because the straw market was not well organised when the datasets were developed	Economic, mass and energy allocation	Not applicable because straw is assumed to be left on the field
Nutrients from straw left on the field		Fertilising effects of crop residues and emissions from the residues are allocated to the crop that generated the residues	Not applicable	Fertilising effects of crop residues are allocated to the crop that generated them (only for P and K). The amount of fertilisers is corrected for the amount of nutrients in crop residues. Allocation of emissions from crop residues is not described in the database report, thus, they are likely allocated to the crop that generated them.
Crop rotation modelling	Phosphorus (P) and potassium (K) input and emission allocation	P and K fertiliser production and emissions due to their application are allocated to each crop proportional to crop exports	Not reported	P and K supplied to the field by cropresidues are allocated to the crop that generated them
	Nitrogen (N) input and emission allocation	Organic N available for the crop to which the fertiliser is applied is allocated to that crop. The remaining fraction that increases the stock of organic matter is allocated to all crops in the rotation. Mineral N is allocated completely to the crop to which it is applied.	Not reported	Allocation not performed

Concerning system boundaries, production and maintenance of infrastructure and machinery are excluded from Agri-footprint datasets because they generally contribute little to LCA results, while PPP and seed production are excluded due to a lack of data at the time when the database was released (grey boxes in Fig. 2) (Blonk Agri-footprint BV, 2014a). In contrast, these processes are included in AGRIBALYSE and ecoinvent.

Concerning modelling assumptions, the main differences are related to allocation of co-products and allocation of emissions from nutrient input (with reference to crop rotation). The way in which co-products (grain and straw) are allocated (or not) can influence results of the LCIA phase (supplementary material, Figs. S10 and S11).

### 3.2 Foreground system analysis

**Fig. 3:**
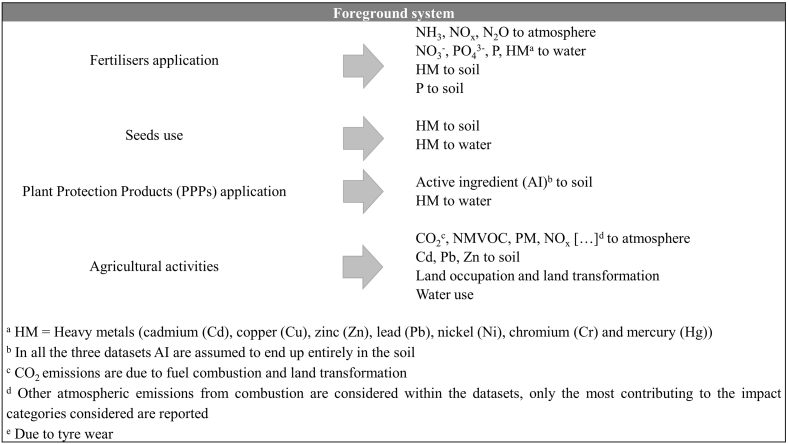
Relation between field activities and environmental emissions and use of resources deduced from analysis of all datasets. Not all activities are considered in all datasets.

Mean crop yields differed slightly among databases (supplementary material, Fig. S1). Only Agri-footprint defines 95% confidence intervals of yields. Relations between field activities and environmental emissions and use of resources were deduced from analysing the datasets (Fig. 3).

#### 3.2.1 Modelling agricultural operations

The databases model agricultural operations for arable crops differently (Table 3). Different reference units for agricultural operations did not allow them to be compared directly, however, we estimated databases' emission factors (kg pollutant/kg diesel) and found differences among them due to differences in the modelling approaches adopted (supplementary material, Table S1). According to Koch and Salou (2015), describing agricultural operations as a number of hours of work in AGRIBALYSE is more flexible than the approach adopted in ecoinvent (operations described as area interested by the operation) because it takes into account different amounts of time required to perform the same process (e.g. tilling different types of soil). Temporal representativeness may strongly influence emission factors of technology-related pollutants, such as particulate matter (EMEP/EEA, 2013): for instance, if machinery is assumed to be older than it really is, emissions of air pollutants due to fuel combustion can be overestimated.

**Table 3:** Decisions made to model agricultural operations in the three databases.

	AGRIBALYSE	Agri-footprint	ecoinvent
Source of data	Operating time and fuel consumption: technical institutes	Not reported	Use of machinery from the GL-Pro project (Nemecek and Baumgartner, 2006)
Temporal and geographical representativeness	2005–2009, France	Not reported	1991–2014 or 2011–2014, World average (GLO)
Reference unit for agricultural operations	Hours of work	Energy content of the fuel consumed	Area
Inclusion of emissions to soil due to tyre wear	Yes	No	Yes

Foreground emissions due to agricultural operations are related to fuel combustion and tyre wear. Fuel combustion includes the compounds emitted in the atmosphere during combustion, considered in the three databases: carbon dioxide (CO_2_), carbon monoxide (CO), particulate matter (PM), ammonia (NH_3_), nitrogen oxides (NO_x_), methane (CH_4_), non-methane volatile compounds (NMVOC) and sulphur dioxide (SO_2_). Tyre wear, which emits cadmium (Cd), lead (Pb) and zinc (Zn) to the soil, is included only in AGRIBALYSE and ecoinvent.

#### 3.2.2 Modelling fertiliser application and nutrient fate

Three elements characterise the modelling approach adopted for fertiliser application and nutrient fate: (i) amount of nutrients provided to the field, (ii) type of fertilisers used (“fertiliser mix”) and (iii) models adopted for nutrient loss to the environment. Data sources for amounts of nutrients applied to the soil vary (Table 2). Specifically, data used for Agri-footprint are derived from Feedprint reports (Vellinga et al., 2013), mainly based on personal communications (Blonk Agri-footprint BV, 2014b). Amounts of phosphorus and potassium applied vary and no nitrogen fertiliser is applied to pea in ecoinvent (supplementary material, Fig. S2). AGRIBALYSE allocates fertilisers applied within a crop rotation, whereas Agri-footprint and ecoinvent do not appear to do so (Table 2). Different kinds and amounts of fertilisers are included in the datasets analysed.

The “fertiliser mix” used in AGRIBALYSE reflects French statistics on fertiliser use from 2005 to 2009 from UNIFA (French fertiliser industry association). Data in Agri-footprint come from international statistics from the International Fertiliser Industry Association (IFA) for 2012, and those in ecoinvent are from the GL-Pro project (Nemecek and Baumgartner, 2006).

Fertiliser application emits nutrients to the environment in the form of nitrogen, phosphorus and potassium compounds and may also emit HMs (Brentrup et al., 2004). Furthermore, application of urea and lime generates emissions of CO_2_ (IPCC, 2006). The three databases do not consider emissions of potassium compounds, however, emissions of nitrogen and phosphorus compounds are included and estimated using different approaches (supplementary material, Table S2).

Relative emissions of NH_3_, expressed as percentage of nitrogen applied to the field through fertilisers emitted to the atmosphere in form of NH_3_, are similar for AGRIBALYSE and ecoinvent but higher for Agri-footprint. Relative emissions of N_2_O and NO_3_^−^ are similar for all databases, except for pea in AGRIBALYSE, for which a higher value was observed. NO_x_ relative emissions differ significantly among the datasets (supplementary material, Fig. S3).

NH_3_ emissions are estimated for AGRIBALYSE and ecoinvent by considering characteristics of fertilisers, as indicated respectively by EMEP/EEA (2009) and the Agrammon model (Agrammon Group, 2009), which is also based on EMEP/EEA (2009) methodology (Nemecek et al., 2014). In contrast, Agri-footprint makes a rougher estimate by using emission factors of the IPCC (2006) for NH_3_ that volatilises after mineral and organic nitrogen fertiliser application. Estimates for Agri-footprint are more conservative than those of AGRIBALYSE and ecoinvent (Fig. S3a).

All databases estimate direct and indirect emissions of N_2_O according to the same method (IPCC, 2006). Relative emissions of N_2_O are the same for all databases, except for pea in AGRIBALYSE, for which the relative emissions is 5 percentage points higher than those of the others (Fig. S3b). NO_x_ emissions are considered only in AGRIBALYSE and ecoinvent, each using a different modelling approach (respectively EMEP/EEA (2009) and NO_x_ emissions = 0.21 × N_2_O emissions), which leads to a higher relative emission for AGRIBALYSE. However, in both databases, NO_x_ emissions represent only a minor (<1%) loss of the nitrogen applied to the field (Fig. S3c). NO_3_^−^ emissions are estimated in AGRIBALYSE according to a model specific to France that considers information about farming practices (e.g. residue management, use of intermediate crops, application of nitrogen fertilisers), crops in the rotation, soil properties and climatic conditions (Koch and Salou, 2015). In Agri-footprint, the average emission factor for NO_3_^−^ emissions of the IPCC (2006) is applied, whereas in ecoinvent the SALCA-NO_3_ model (Richner et al., 2014) is used. For pea datasets in AGRIBALYSE and ecoinvent higher relative NO3^−^ emissions than the amount of nitrogen applied to the field. For the other crops, Agri-footprint had higher NO_3_^−^ relative emissions than AGRIBALYSE, whereas no significant differences were observed between those of ecoinvent and AGRIBALYSE or ecoinvent and Agri-footprint (Fig. S3d).

Three pathways are considered for phosphorus emissions: (i) leaching to groundwater, (ii) runoff and (iii) emission to surface water due to soil erosion. AGRIBALYSE and ecoinvent estimate leaching and runoff using the SALCA-P model (Prasuhn, 2006), validated for Switzerland but not for France, that considers parameters such as soil characteristics and fertiliser type (Nemecek, 2013), whereas Agri-footprint assumes that the amount of phosphorous in fertilsers and manure is emitted to the soil and uses a fixed emission factor to estimate the fraction of phosphorous that reaches freshwater (Blonk Agri-footprint BV, 2014b). AGRIBALYSE and ecoinvent include emissions of phosphorous due to soil erosion as considered by Prasuhn (2006), whereas Agri-footprint does not include it due to limited data availability (Blonk Agri-footprint BV, 2014a). CO_2_ emissions from urea and lime application are included in all three databases, which use the same emission factors (IPCC, 2006).

#### 3.2.3 Modelling PPP application and environmental fate

PPP use is modelled according to several data sources (Table 2). PPP application and fate modelling have large uncertainties. Indeed, Agri-footprint documentation emphasises using default data and suggests that dataset users modify the inventory with primary data whenever possible. Many PPPs are included in the datasets, even though AGRIBALYSE and ecoinvent lack specific production process for some of them, for which they use average PPP production inventories.

Regarding PPP fate, all databases assume that 100% of PPPs end up in agricultural soil after application. This is considered a highly controversial assumption when estimating the contribution of PPPs to toxicity impacts due to possible different initial distribution of PPPs (Rosenbaum et al., 2015). Another problem is the representativeness of the PPPs included in the datasets. PPP use in France is subject to European Union legislation requiring that active ingredients are approved before being sold on the market (EU, 2009). However, some active ingredients in the datasets, such as bitertanol (AGRIBALYSE and ecoinvent) and metolachlor (Agri-footprint), are no longer authorised in France (EC, 2015) (supplementary material, Table S3) and should be excluded from the inventory of any crop cultivation in France.

#### 3.2.4 Modelling HM input, mass balance and fate

HMs mass balance is performed in all datasets following the same principle (Freiermuth, 2006). HMs emitted to the soil are calculated as the sum of all HMs that enter the agricultural system (due to seeds, fertilisers, pesticides, fertilisers, manure and atmospheric deposition) minus the sum of all HMs that leave it (due to leaching, erosion and biomass removal). Estimates of HM flows to and from the soil are highly uncertain. In fact, some datasets for crop production estimate that more HMs leave the system than enter it, resulting in a net decrease of HMs in the soil. As emphasised by Koch and Salou (2013), these figures should not be interpreted as true removal of HMs from the soil, but rather as an effect of uncertainty in input and output data.

Atmospheric deposition and application of mineral and organic fertilisers represent the major sources of HM inputs to agricultural soil (Nicholson et al., 2003) and are considered in all datasets. In contrast, other HM sources, such as PPPs and seeds, are considered only in AGRIBALYSE and ecoinvent. Leaching and exportation in biomass are considered as removal mechanisms for HMs in the soil in all datasets, whereas emissions of HMs to water through erosion of soil particles is included only in AGRIBALYSE and ecoinvent.

Among the three databases, different literature data are used to estimate amounts of HMs input to and removed from soil. HM removal due to leaching is estimated according to available average data and all three databases use the same values. Since specific data for France were not available, data for Switzerland were used in AGRIBALYSE (Koch and Salou, 2013). AGRIBALYSE and ecoinvent estimate soil erosion using the same equation, however, estimated soil HM content and amount of soil eroded differ (supplementary material, Table S5). Another source of emissions of HMs to soil is tyre wear due to agricultural operations (see section 3.2.1).

#### 3.2.5 Modelling irrigation

**Fig. 4:**
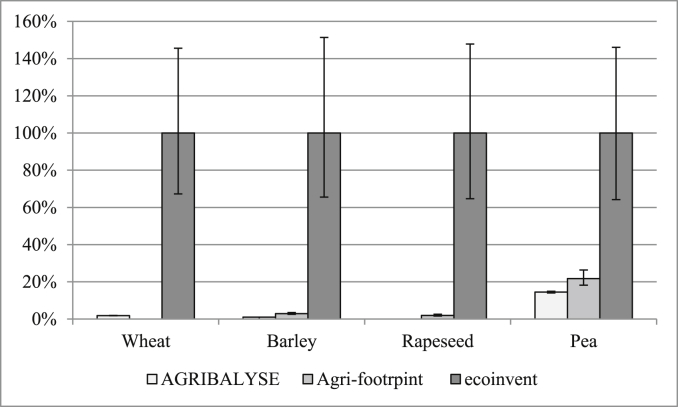
Relative mean water irrigation volumes per hectare of cultivated land and 95% confidence intervals. For each arable crop, the maximum irrigation volume is reported as 100% and the others are expressed as percentages of the maximum.

Irrigation volumes for a given crop vary greatly among the datasets (Fig. 4), according to the data source: AGRIBALYSE data were collected from technical institutes, Agri-footprint data were taken from the “blue water footprint” of Mekonnen and Hoekstra (2010), and ecoinvent data came from the work by Doll and Zhang (2010). Ecoinvent has the highest irrigation volumes for each crop. Furthermore, different elementary flows are used to model irrigation: “Water, river” in AGRIBALYSE, “Water, unspecified natural origin, FR” in Agri-footprint, and 55% “Water, river FR” and 45% “Water, well, in ground, FR” in ecoinvent. Differences in the types and locations of water sources included in the inventory may lead to large differences in predictions of water depletion after characterisation, especially if characterisation factors are spatially explicit.

#### 3.2.6 Modelling land occupation and transformation

All databases consider agricultural land occupation as m^2^y, taking into account the duration of cultivation. Land transformation is modelled according to different approaches (Table 4). Ecoinvent assumes zero net land transformation because it considers that no land is transformed for arable crops in France (Nemecek et al., 2014). In contrast, AGRIBALYSE and, for wheat and rapeseed, Agri-footprint, include land transformation from natural areas, such as pasture and forests, or from permanent crops to agricultural land uses. Furthermore, AGRIBALYSE considers transformation from “discontinuously built urban” land uses to agricultural land uses.

**Table 4:** Models and sources of data for land transformation.

	AGRIBALYSE	Agri-footprint	ecoinvent
Model	Frischknecht and Jungbluth, 2007	Direct Land Use Change Assessment Tool (Blonk Consultants, 2014)	Milà I Canals et al., 2012 (see Nemecek et al., 2014)
Source of data	Teruti-Lucas, 2006	Direct Land Use Change Assessment Tool based on data from FAOstat (FAO, 2012)	FAOstat (FAO, 2012) (see Nemecek et al., 2014)

Land transformation may imply emissions of CO_2_ due to organic carbon mineralisation. These emissions are included only in Agri-footprint, which estimated them using the Direct Land Use Change Assessment Tool, compliant with the PAS 2050-1 (BSI, 2012) and European environmental footprint (EF) methods (Blonk Consultants, 2014; EC, 2013), assuming that the previous land use was not known. AGRIBALYSE excludes greenhouse gas (GHG) emissions from land use change due to lack of data about land occupation over time (Koch and Salou, 2013).

### 3.3 Life cycle impact assessment results

**Fig. 5:**
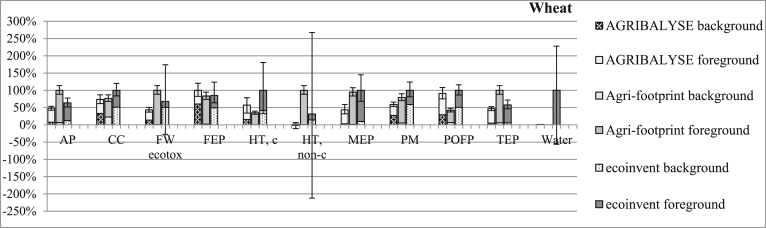
Relative foreground and background contributions (mean and 95% confidence interval) per kg of wheat grain. For each impact category, the largest value is reported as 100% and the others are expressed as percentages of the maximum. The following impact categories were considered: acidification (AP), climate change (CC), freshwater ecotoxicity (FW ecotox), freshwater eutrophication (FEP), human toxicity-cancer (HT, c), human toxicity-non cancer (HT, non-c), marine eutrophication (MEP), particulate matter (PM), photochemical ozone formation (POFP), terrestrial eutrophication (TEP), water resource depletion (Water).

**Fig. 6:**
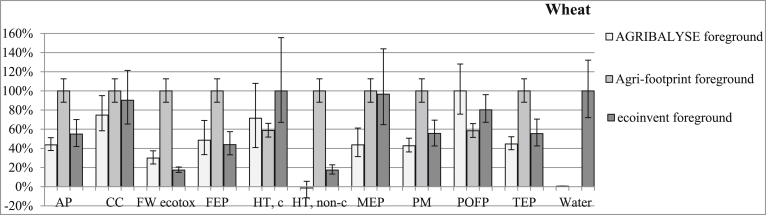
Relative foreground system contributions (mean and 95% confidence interval) per kg of wheat grain. For each impact category, the largest value is reported as 100% and the others are expressed as percentages of the maximum. The following impact categories were considered: acidification (AP), climate change (CC), freshwater ecotoxicity (FW ecotox), freshwater eutrophication (FEP), human toxicity-cancer (HT, c), human toxicity-non cancer (HT, non-c), marine eutrophication (MEP), particulate matter (PM), photochemical ozone formation (POFP), terrestrial eutrophication (TEP), water resource depletion (Water).

In general, the choice of the database (and related dataset) used to model a given product can lead to different LCIA results (Fig. 5 and Fig. S4). In some cases (e.g. toxicity-related impact categories and water depletion in ecoinvent), uncertainty in results from a given dataset is larger than differences in results among the databases. This high degree of uncertainty can affect interpretation of results and the ability to achieve the goal of the study. In other cases, results differ greatly even when considering the uncertainty. This is particularly true for some impact categories, i.e. acidification and terrestrial eutrophication, when the analysis focuses only on the foreground system (Fig. 6 and Fig. S5). Thus, the contribution of background datasets can sometimes partly offset differences between LCIAs due to different modelling approaches.

**Fig. 7:**
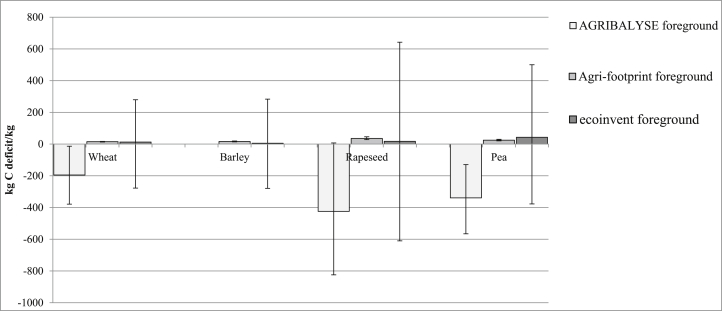
Mean potential land use impacts of the foreground system and 95% confidence intervals per kg of arable crop.

Fig. 5 reports the LCIA for wheat, showing foreground and background contributions. LCIAs for the other three arable crops tested are reported in the supplementary material (Fig. S4). Only the impact categories which contributed to the foreground system are reported in Fig. 5, Fig. 6. Three impact categories (ionising radiation; mineral, fossil and renewable resource depletion; and ozone depletion potential) were excluded from the analysis because they were influenced only by the background system. The impact of the foreground system on land use is reported in Fig. 7.

Agri-footprint generally estimated lower contributions from the background system than the other databases, which could be explained by its smaller system boundaries for background systems than those of AGRIBALYSE and ecoinvent (Fig. 2). When considering the effects of allocation in Agri-footprint, large differences were observed between datasets with no allocation and those with allocation (supplementary material, Figs. S10 and S11).

Concerning the foreground system (Figs. S6 and S5), a significant difference in climate change impact was observed only for rapeseed, due to Agri-footprint's inclusion of CO_2_ emissions from land transformation and use of more nitrogen fertilisers, which increased N_2_O emissions.

The main contributors in the foreground system to the impact categories particulate matter, photochemical ozone formation, acidification and terrestrial eutrophication were NH_3_ and NO_x_ emissions. In contrast, marine eutrophication and freshwater eutrophication were influenced mainly by emissions to water of NO_3_^−^ and phosphorus compounds, respectively.

Agri-footprint predicted significantly higher foreground contributions than AGRIBALYSE and ecoinvent to acidification, particulate matter and terrestrial eutrophication, mainly due to NH_3_ emissions. AGRIBALYSE and ecoinvent showed no significant differences between acidification, particulate matter and terrestrial eutrophication of wheat and rapeseed, but did so for pea, due to significant differences in nitrogen fertiliser application and, therefore, NH_3_ emissions.

Photochemical ozone formation was caused mainly by NO_x_ emitted by application of nitrogen fertilisers and combustion of diesel for agricultural machinery. Agri-footprint datasets for wheat, rapeseed and pea had significantly lower photochemical ozone formation because they excluded NO_x_ emissions from nitrogen fertiliser application. Agri-footprint and ecoinvent had significantly higher marine eutrophication for wheat than AGRIBALYSE, and Agri-footprint had higher marine eutrophication than AGRIBALYSE for rapeseed, due to higher NO_3_^−^ emissions per unit of nitrogen fertiliser applied to the field.

Emission of HMs and PPPs were the main contributors to the human-toxicity-related impact categories in the foreground system. Human toxicity was mainly influenced by HM emissions. As in the inventory, negative contributions were predicted due to uncertainty in the modelling rather than a positive potential impact on human health (Koch and Salou, 2013). The impact “human toxicity, cancer” (influenced mainly by chromium emissions to water) for wheat was higher in ecoinvent than in Agri-footprint, whereas for pea it was highest in Agri-footprint. Significant differences in “human toxicity, non-cancer” were also observed for wheat, barley and pea.

PPP emissions to the soil had a large influence on freshwater ecotoxicity, however, characterisation factors for some PPP assumed to be released to the soil were missing in the chosen characterisation model (supplementary material, Table S4), which may underestimate PPP impacts. For irrigation, differences in irrigation volumes and water sources explained significant differences in potential water depletion among the databases. Since the ILCD characterisation model for water was spatially-explicit, characterisation factors of elementary flows used in Agri-footprint and ecoinvent were nearly four times as high as the ones used in AGRIBALYSE.

AGRIBALYSE predicted a negative land use impact because it assumed transformation from discontinuously built urban soil to agricultural soil, which resulted in a strong negative contribution of the foreground system (Fig. 7). Indeed, the ILCD characterisation model associated a highly negative characterisation factor with this transformation. In contrast, Agri-footprint and ecoinvent predicted a similar average land use impact, although land use and land transformation are modelled differently.

## 4 Discussion

Analysis of secondary datasets for arable crops highlighted significant differences among the life cycle impacts that are influenced by sources of activity data and modelling approaches adopted to estimate environmental emissions and use of resources, such as land use. Generally, different arable crops are modelled in a similar way within a given database, whereas greater differences were observed for the same arable crop modelled in different databases. Here we provide an overview of main features of the databases analysed and considerations about their accuracy.

### 4.1 Data characteristics

Databases differ in their sources of data: AGRIBALYSE and Agri-footprint use average data for France, whereas ecoinvent uses data from one French region to represent all of France. In AGRIBALYSE and ecoinvent, data uncertainty is assessed for each input from and output to the foreground system, except for crop yields, using the pedigree matrix approach. In contrast, Agri-footprint estimates uncertainty in crop yields and derives uncertainty in input flows and emissions accordingly. For this reason, the range of the 95% confidence interval of LCIA varied among impact categories for AGRIBALYSE and ecoinvent but not for Agri-footprint.

### 4.2 System boundary definition

The choice of system boundaries influences contributions of the background system to results. Indeed, Agri-footprint datasets – for which agricultural infrastructure, machinery, and production of PPP and seeds are excluded from system boundaries – had generally lower contribution from the background system than the others for nearly all impact categories analysed. System boundaries of secondary data should be consistent with the goal and scope of the study and, when pertinent, with LCA guidelines, which sometimes have instructions for including or excluding specific processes. The inclusion of infrastructure, for example, can become important depending upon the impact categories analysed (Frischknecht et al., 2007). Moreover, if the aim of the LCA is to assess environmental burdens of a product to comply, for example, with the EF, then, according to the EF guide (EC, 2016), infrastructure and machinery should be included within system boundaries.

### 4.3 Agricultural practice modelling

Management of an agricultural system includes complex dynamics that should be considered when performing a LCA. Indeed, management of agricultural residues and composition of a crop rotation can affect field productivity and the inputs required (Cherubini and Ulgiati, 2010, Nemecek et al., 2015). The three databases modelled these effects using different approaches (Table 2). Furthermore, allocating the impact to co-products and the choice of allocation method can influence the LCIA strongly (supplementary material, Fig. S10, Fig. S11). Although the most appropriate way to model agricultural practices remains under debate (e.g. Cherubini and Ulgiati, 2010, Nemecek et al., 2015), it is important that LCA practitioners verify that modelling of crop rotation and co-product management are consistent with the goal and scope of their studies and, if applicable, product category rules.

### 4.4 Agricultural operation modelling

Agricultural operation modelling differs in the number of operations, the number of passes and the time-related representativeness. Other elements that influence impacts of agricultural operations substantially, such as machine power and soil texture (Lovarelli et al., 2016, Van linden and Herman, 2014), are not explicitly considered in the datasets analysed, and Lovarelli et al. (2016) found that this can lead to misleading results. Use of agricultural machinery causes airborne emissions due to fuel combustion and emissions of HMs due to tyre wear (Hjortenkrans et al., 2007), whose emissions are included only in AGRIBALYSE and ecoinvent.

### 4.5 Fertiliser application and nutrient fate modelling

Significant differences among the datasets were found in the amount of fertilisers applied to the field. Official statistics on amounts of fertilisers per crop were not available for France, therefore it was not possible to check which database contains the most accurate data.

Nutrient fate is greatly influenced by site-specific conditions, such as environmental conditions, soil type, agricultural management practices and fertiliser type (Brentrup et al., 2000). Hence, spatially-explicit modelling of emissions from agricultural systems is considered of paramount importance (Basset-Mens et al., 2006, Biswas et al., 2008, Cederberg et al., 2013). A spatially-explicit approach was partially applied in AGRIBALYSE and ecoinvent but not in Agri-footprint.

Agri-footprint estimates of NH_3_ emissions, based on IPCC guidelines (IPCC, 2006), lead to significantly higher emissions per unit of nitrogen applied to the field than in AGRIBALYSE and ecoinvent, explaining Agri-footprint's higher acidification and terrestrial eutrophication impacts. In contrast, NO_3_^−^ relative emissions were equal for all crops except peas. Relative emissions for NO_x_ from nitrogen fertiliser application were higher in AGRIBALYSE than in ecoinvent and were not considered in Agri-footprint.

Databases expressed phosphorus compound emissions using different flows, limiting the ability to compare inventory data. Scherer and Pfister (2015) found that estimates of emissions of phosphorus compounds in ecoinvent were up to one order of magnitude lower than results of their model. Phosphorus compound emissions represent the main contribution to freshwater eutrophication, which, except for peas, did not significantly differ among the databases, despite significant differences in phosphorus fertiliser application.

### 4.6 PPP application and environmental fate modelling

Estimation of PPP emission and fate is a topic of intense discussion both in the LCA community and beyond (Rosenbaum et al., 2015). In the three databases, active ingredients were assumed to end up completely in the soil after application. However, depending on the active ingredient, application method, weather and soil conditions, crop characteristics and irrigation, PPP fate can change, and using a pre-determined fate factor can lead to extremely high uncertainty (Rosenbaum et al., 2015).

Moreover, the databases analysed included active ingredients no longer authorised in France, of which one (carbendazim) contributes most to the freshwater ecotoxicity impact of wheat in Agri-footprint. When choosing a dataset, it is, therefore, recommended to verify that modelling of PPP application follows legislation of the country in question. Regarding the LCIA, PPP emissions influenced freshwater ecotoxicity and, to a lesser extent, human toxicity. However, estimated impacts can be influenced by assumptions about PPP fate and the type of PPP used. Some PPP emissions did not have associated characterisation factors in the ILCD characterisation model, which may have caused toxicity-related impacts to be underestimated. Therefore, since none of the characterisation methods and related models currently available has characterisation factors for all possible emissions of PPPs, LCA practitioners should be aware that the combination of characterisation model and dataset can influence results of toxicity-related impact categories.

### 4.7 Heavy metal inputs and environmental fate modelling

Mass balance and fate of HMs is affected by several uncertainties and limitations. For example, in the datasets analysed, uncertainty in HM inputs to the agricultural system and in fate modelling led to misleading negative emissions to the agricultural soil (Koch and Salou, 2013) that resulted in negative contributions to human toxicity and freshwater ecotoxicity impacts. Furthermore, the choice of characterisation model can influence assessment of impacts on human health greatly (Pizzol et al., 2011).

### 4.8 Irrigation modelling

Databases differ in both irrigation volumes and water flows, with different spatially-explicit characterisation factors, which contribute to different LCIA results. In contrast, temporal variability in irrigation is not considered in any of the datasets analysed. Even though Pfister and Bayer (2014) highlighted the importance of considering temporal variability when assessing the impact of water stress, databases currently implemented in commonly used LCA software do not report temporally-explicit flows. Therefore, LCA practitioners are recommended to prefer spatially-explicit water flows to assess water resource depletion, while LCA database developers should focus on including temporal variability in water withdrawals in their datasets.

### 4.9 Land transformation modelling

The three databases model land transformation differently. In Agri-footprint and AGRIBALYSE, in which average data are considered, land transformation is reported, but different amounts of transformed land are considered. In contrast, ecoinvent excludes net land transformation. Transformation from a discontinuously built urban area to an agricultural one gives a relevant negative contribution to land use impact in AGRIBALYSE. CO_2_ emissions due to land transformation are considered relevant and are included only in Agri-footprint. In addition, impact due to different land management practices are currently difficult to assess.

## 5 Conclusions

Datasets from different databases that model the same crop have methodological differences that can lead to significantly different LCIA results. In the present study nine relevant elements of datasets that model arable crop cultivation were analysed to highlight similarities and differences and investigate the extent to which they affect results. The nine elements are data sources, system boundary definition, agricultural practice modelling, agricultural operation modelling, fertiliser application and nutrient fate modelling, PPP application and environmental fate modelling, HM input and environmental fate modelling, irrigation modelling and land transformation modelling.

Results of the present study provide LCA practitioners with elements they can use to evaluate characteristics of datasets which they use for modelling (not only the databases analysed here), to choose the most appropriate one depending on the aim and scope of the study, and to interpret results. Furthermore, to a certain extent, they can provide information to database developers to improve dataset quality. For instance, the exclusion of infrastructure, machinery, and production of PPPs and seeds from system boundaries can significantly influence contribution of the background system to nearly all impact categories.

Activity data from which datasets were built differ greatly because datasets rely on different data sources. Since official statistics on arable crop production in France are currently not available for most activity data, it was not possible to identify the most accurate datasets, however, a check of activity data by a pool of experts may yield a higher level of accuracy.

Concerning the LCIA results, the foreground system contributed more to overall impact for most impact categories and nearly all of the datasets analysed. Impacts of the foreground system were associated mainly with field emissions, most of which are estimated with models. Since field emissions are influenced largely by site-specific conditions, including site-specific parameters in the modelling may lead to more accurate estimates.

Although the present study examined only 12 datasets modelling four arable crops, we consider the present work as a basis from which to start analysing and interpreting other datasets of agricultural products. Furthermore, as the study highlighted that much of the LCIA is associated with estimated emissions, we ask other researchers to explore the pertinence of models used to estimate field emissions and to provide more details about the representativeness of and uncertainty in the results.
