# Supplementary material for: Systematic analysis of secondary life cycle inventories when modelling agricultural production: A case study for arable crops
Source: J Clean Prod. 2018 Jan 20;172:3990–4000. doi: 10.1016/j.jclepro.2017.03.179 (PMC5750820; doi:10.1016/j.jclepro.2017.03.179)
Supplement: Supplementary file 1 [file mmc1.docx]

**Supplementary material**

Systematic analysis of secondary life cycle inventories when modelling agricultural production: A case study for arable crops

Sara Corrado^1^, Valentina Castellani^2^, Luca Zampori^2^, Serenella Sala^2^

1 Università Cattolica del Sacro Cuore, Istituto di Chimica Agraria e Ambientale, Via Emilia Parmense 84, 29122 Piacenza (PC), Italy

^2^ European Commission, Joint Research Centre, Directorate D – Sustainable Resources – Bio-Economy Unit, via Enrico Fermi 2749 TP290, I-21027 Ispra (Va), Italy

Corresponding author: Valentina Castellani, [valentina.castellani@ec.europa.eu](mailto:valentina.castellani@ec.europa.eu)

**Content**

- Average crop yields
- Agricultural operations
- Modelling of fertilisers application and nutrients fate
- Modelling of plant protection products and their environmental fate
- Modelling of soil erosion
- LCIA
- Effects of modelling approach on the LCIA
- Effects of allocation on the LCIA
- References

**Average crop yields**

Figure S1 reports the crop yield assumed in each dataset.

Figure S1: Average crop yields with 95% confidence interval. The 95% confidence interval of the yields is defined only in Agri-footprint datasets, not in AGRIBALYSE and ecoinvent datasets

**Agricultural operations**

Table S1 reports the average emissions factors for agricultural machineries expressed in terms of mass of pollutant emitted by the combustion of 1 kg of diesel.

Table S1: Emissions factors expressed in term of mass of pollutant emitted by a unit of mass of diesel (kg/kg_diesel_)

|  | **AGRIBALYSE** | **Agri-footprint** | **ecoinvent** |
| --- | --- | --- | --- |
| **NH_3_** | 2.0E-05 | 1.0E-05 | 2.0E-05 |
| **PM < 2.5 um** | 4.3E-03 | 1.4E-03 | 4.7E-03 |
| **CO_2_** | 3.1 | 3.2 | 3.1 |
| **N_2_O** | 1.2E-04 | 2.6E-05 | 1.2E-04 |
| **CH_4_** | 1.3E-04 | 1.1E-04 | 1.3E-04 |
| **CO** | 5.4E-03 | 4.9E-03 | 8.1E-03 |
| **NOx** | 4.2E-02 | 3.0E-02 | 4.5E-02 |
| **NMVOC** | 2.7E-03 | 2.8E-03 | 2.7E-03 |

**Modelling of fertilisers application and nutrients fate**

In Figure S1 the amount of nitrogen, phosphorus and potassium fertilisers applied to the field for the arable crops cultivation is reported.

Table S2 reports the modelling approaches adopted in the three datasets to estimate the nutrients fate and Figure S2 represents the resulting emissions of nitrogen compounds expressed as percentage of nitrogen applied to the field in each dataset.

|  |
| --- |
|  |
| AGRIBALYSE Agri-footprint ecoinvent |
|  |

Figure S2: Average fertilisers application and 95% conference intervals per ha of field. The 95% confidence interval is not reported in the AGRIBALYSE barley dataset, N refers to nitrogen fertilisers as N, P to phosphorus fertilizers as P_2_O_5_ and K to potassium fertilisers as of K_2_O. For each nutrient, the highest result among the three datasets is reported as 100% and the others are expressed as percentage of the maximum amount.

Table S2: Models adopted for nutrients fate

|  | **AGRIBALYSE** | **Agri-footprint** | **ecoinvent** |
| --- | --- | --- | --- |
| Ammonia (NH_3_) | (EMEP/EEA, 2009) Tier 2 (for organic fert.); EMEP/CORINAIR 2006 Tier 2 (see (Koch and Salou, 2013) (for mineral fert.) | (IPCC, 2006) | (Asman, 1992) (for mineral fertiliser) |
| Nitrogen oxides (NO_x_) | (EMEP/EEA, 2009) Tier1 | Not considered | Personal communication in (Nemecek and Schnetzer, 2011) |
| Nitrate (NO_3_^-^) | COMIFER 2001 adjusted (see (Koch and Salou, 2013) | (IPCC, 2006) (all the N leached is emitted as NO3) | SALCA-NO3 (Richner et al., 2014) |
| Dinitrogen oxide (N_2_O) (direct + indirect emissions) | (IPCC, 2006) Tier 1 | (IPCC, 2006) Tier 1 | (IPCC, 2006) Tier 1 |
| Phosphorus (P) (leaching) | SALCA-P (Prasuhn, 2006) | 0,05 of P in fertilisers and manure reaches freshwater | SALCA-P (Prasuhn, 2006) |
| Phosphorus (P) (runoff) | SALCA-P (Prasuhn, 2006) |  | SALCA-P (Prasuhn, 2006) |
| Phosphorus (P) (erosion) | SALCA – P (Prasuhn, 2006) | Not included due to limited data availability | SALCA-P (Prasuhn, 2006) |

|  |  |
| --- | --- |
| AGRIBALYSE Agri-footprint ecoinvent |  |

Figure S3: Average emissions of N compounds and 95% confidence interval, expressed as percentage ratio of the N emitted in the environment to the N applied to the field through fertilisers

**Modelling of plant protection products and environmental fate**

In Table S3 a list of the active ingredient that are included in datasets, whose use was not authorised in France anymore, according to European legislation, is reported.

Table S3: Active ingredients included in the analysed datasets whose use is not authorised in France

| **Active ingredient** | **Database** |
| --- | --- |
| Anthraquinone | ecoinvent |
| Bitertanol | AGRIBALYSE, ecoinvent |
| Carbendazim | Agri-footprint, ecoinvent |
| Choline chloride | AGRIBALYSE, ecoinvent |
| Cyfluthrin | AGRIBALYSE |
| Flusilazole | ecoinvent |
| Ioxynil | AGRIBALYSE |
| Metolachlor | Agri-footprint |
| Oxydemeton methyl | AGRIBALYSE |
| Procymidone | ecoinvent |
| Trifluralin | AGRIBALYSE, ecoinvent |
| Vinclozolin | Agri-footprint, ecoinvent |

In Table S4 the active ingredients emitted in the environment for which a characterisation factor did not exist for any of the impact categories considered are reported.

Table S4: List of emitted active ingredients of PPP for which a characterisation factor was not reported for any of the impact categories considered. X indicates that the emissions of the specific active ingredient were considered in the database

|  | **AGRIBALYSE** | **Agri-footprint** | **ecoinvent** |
| --- | --- | --- | --- |
| Boscalid | X |  |  |
| Fenpropidin |  |  | X |
| Fenpropimorph |  |  | X |
| Florasulam | X |  | X |
| Fluoxastrobin | X |  |  |
| Flupyrsulfuron-methyl |  |  | X |
| Fluquinconazole | X |  |  |
| Iodosulfuron | X |  |  |
| Iodosulfuron-methyl-sodium |  |  | X |
| Mefenpyr-diethyl | X |  |  |
| Mesosulfuron-methyl (prop) | X |  | X |
| Metaldehyde | X |  | X |
| Metconazole | X |  | X |
| Metosulam |  |  | X |
| Picoxystrobin | X |  | X |
| Prohexadione-calcium |  |  | X |
| Propoxycarbazone-sodium (prop) |  |  | X |
| Prothioconazol | X |  |  |
| Pyraclostrobin (prop) | X |  | X |
| Silthiofam |  |  | X |
| Spiroxamine |  |  | X |
| Trifloxystrobin |  |  | X |

**Modelling of soil erosion**

Table S5 reports the references used to model soil erosion in the AGRIBALYSE and ecoinvent datasets.

Table S5: Equation used to estimate the heavy metal and phosphorus emissions due to erosion and sources of data and parameters

| **M_erosion_**  **(heavy metals emissions through erosion)** | **=** | **Ctot**  **(HM content in the soil)** | ***** | **B**  **(soil erosion)** | ***** | **A**  **(accumulation factor)** | ***** | | **F**  **(fraction of soil the reaches rivers)** |
| --- | --- | --- | --- | --- | --- | --- | --- | --- | --- |
| ***Value/sources of data*** | | | | | | | | | |
| **AGRIBALYSE v1.2** | BDAT database (RMQS, 2013) | | RUSLE soil loss equation (USDA – Agricultural Research Service, 2005) | | 1.86  (Freiermuth, 2006) | | | 0.2  (Freiermuth, 2006) | |
| **ecoinvent v3.1** |  | (Keller and A., 2001) |  | (Oberholzer et al., 2001) |  | 1.86 | |  | 0.2 |

**LCIA**

In this section the LCIA for 1 kg of the analysed arable crops is reported. In each figure, the result for land use is not displayed because it is out of scale. Figure S4 represents the LCIA by showing the foreground and background contributions, whereas Figure S5 represents only the foreground contribution. In both Figure S4 and Figure S5 the 95% confidence interval is reported.

|  |
| --- |
|  |
|  |
|  |

Figure S4: Average foreground and background systems contributions to LCIA and 95% confidence interval for 1 kg of the analysed arable crops. For each impact category, the higher result is reported as 100% and the other are expressed as percentage of the maximum amount. The following impact categories were considered: acidification (AP), climate change (CC), freshwater ecotoxicity (FW ecotox), freshwater eutrophication (FEP), human toxicity, cancer effects (HT,c), human toxicity, non-cancer effects (HT, non-c), marine eutrophication (MEP), particulate matter (PM), photochemical ozone formation (POFP), terrestrial eutrophication (TEP), water resource depletion (Water).

|  |
| --- |
|  |
|  |
|  |

Figure S5: Average foreground system contributions to LCIA and 95% confidence interval for 1 kg of the the analysed arable crops. For each impact category, the higher result is reported as 100% and the other are expressed as percentage of the maximum amount. The following impact categories were considered: acidification (AP), climate change (CC), freshwater ecotoxicity (FW ecotox), freshwater eutrophication (FEP), human toxicity, cancer effects (HT,c), human toxicity, non-cancer effects (HT, non-c), marine eutrophication (MEP), particulate matter (PM), photochemical ozone formation (POFP), terrestrial eutrophication (TEP), water resource depletion (Water).

**Effects of modelling approach on the LCIA**

Figure S6, Figure S7, Figure S8 and Figure S9 report a comparison of average potential impact of 1 kg of the analysed crop at farm gate as modelled in ecoinvent datasets following three modelling approaches: attributional (also called “Alloc def” in ecoinvent database), cut-off system model (also called “Alloc rec” in ecoinvent database) and consequential (also called “Conseq” in ecoinvent database).

Figure S6: Average potential impact of 1 kg of wheat at farm gate as modelled in ecoinvent datasets with different approaches. For each impact category, the higher result is reported as 100% and the other are expressed as percentage of the maximum amount. The following impact categories were considered: climate change (CC), ozone depletion potential (ODP), human toxicity, cancer effects (HT,c), human toxicity, non-cancer effects (HT, non-c), particulate matter (PM), ionising radiation (IR), photochemical ozone formation (POFP), , acidification (AP), terrestrial eutrophication (TEP), freshwater eutrophication (FEP), marine eutrophication (MEP), freshwater ecotoxicity (FW ecotox), water resource depletion (Water), mineral, fossil and renewable resource depletion (Res).

Figure S7: Average potential impact of 1 kg of barley at farm gate as modelled in ecoinvent datasets with different approaches. For each impact category, the higher result is reported as 100% and the other are expressed as percentage of the maximum amount. The following impact categories were considered: climate change (CC), ozone depletion potential (ODP), human toxicity, cancer effects (HT,c), human toxicity, non-cancer effects (HT, non-c), particulate matter (PM), ionising radiation (IR), photochemical ozone formation (POFP), , acidification (AP), terrestrial eutrophication (TEP), freshwater eutrophication (FEP), marine eutrophication (MEP), freshwater ecotoxicity (FW ecotox), water resource depletion (Water), mineral, fossil and renewable resource depletion (Res).

Figure S8: Average potential impact of 1 kg of rapeseed at farm gate as modelled in ecoinvent datasets with different approaches. For each impact category, the higher result is reported as 100% and the other are expressed as percentage of the maximum amount. The following impact categories were considered: climate change (CC), ozone depletion potential (ODP), human toxicity, cancer effects (HT,c), human toxicity, non-cancer effects (HT, non-c), particulate matter (PM), ionising radiation (IR), photochemical ozone formation (POFP), , acidification (AP), terrestrial eutrophication (TEP), freshwater eutrophication (FEP), marine eutrophication (MEP), freshwater ecotoxicity (FW ecotox), water resource depletion (Water), mineral, fossil and renewable resource depletion (Res).

Figure S9: Average potential impact of 1 kg of pea at farm gate as modelled in ecoinvent datasets with different approaches. For each impact category, the higher result is reported as 100% and the other are expressed as percentage of the maximum amount. The following impact categories were considered: climate change (CC), ozone depletion potential (ODP), human toxicity, cancer effects (HT,c), human toxicity, non-cancer effects (HT, non-c), particulate matter (PM), ionising radiation (IR), photochemical ozone formation (POFP), acidification (AP), terrestrial eutrophication (TEP), freshwater eutrophication (FEP), marine eutrophication (MEP), freshwater ecotoxicity (FW ecotox), water resource depletion (Water), mineral, fossil and renewable resource depletion (Res).

**Effects of allocation on the LCIA**

Figure S10 and Figure S11 report the LCIA of the average potential impact of 1 kg of wheat and barley at the farm gate, considering the effect of allocation of the impact to co-products for the Agri-footprint datasets. Particularly, four allocation criteria are reported for Agri-footprint datasets: impact allocated entirely to the grains (no alloc) and allocation according to the economic value, the energy content and the mass.

Figure S10: LCIA of 1 kg of wheat at farm gate. In AGRIBALYSE and ecoinvent the impact is entirely allocated to the grains, in Agri-footprint different allocation criteria are considered: economic, energy and mass. Agri-footprint_no alloc refers to the datasets considered in the present study, in which the entire impact of cultivation is allocated to grains. For each impact category, the higher result is reported as 100% and the other are expressed as percentage of the maximum amount. The following impact categories were considered: climate change (CC), ozone depletion potential (ODP), human toxicity, cancer effects (HT,c), human toxicity, non-cancer effects (HT, non-c), particulate matter (PM), ionising radiation (IR), photochemical ozone formation (POFP), acidification (AP), terrestrial eutrophication (TEP), freshwater eutrophication (FEP), marine eutrophication (MEP), freshwater ecotoxicity (FW ecotox), water resource depletion (Water), mineral, fossil and renewable resource depletion (Res).

Figure S11: LCIA of 1 kg of barley at farm gate. In AGRIBALYSE and ecoinvent the impact is entirely allocated to the grains, in Agri-footprint different allocation criteria are considered: economic, energy and mass. Agri-footprint_no alloc refers to the datasets considered in the present study, in which the entire impact of cultivation is allocated to grains. For each impact category, the higher result is reported as 100% and the other are expressed as percentage of the maximum amount. The following impact categories were considered: climate change (CC), ozone depletion potential (ODP), human toxicity, cancer effects (HT,c), human toxicity, non-cancer effects (HT, non-c), particulate matter (PM), ionising radiation (IR), photochemical ozone formation (POFP), acidification (AP), terrestrial eutrophication (TEP), freshwater eutrophication (FEP), marine eutrophication (MEP), freshwater ecotoxicity (FW ecotox), water resource depletion (Water), mineral, fossil and renewable resource depletion (Res).

**References**

Asman, W.A.H., 1992. Ammonia emissions in Europe: updated emission and emission variations. Report no. 228471008.

EMEP/EEA, 2009. Air pollutant emission inventory guidebook.

Freiermuth, R., 2006. Modell zur Berechnung der Schwermetallflüsse in der Landwirtschaftlichen Ökobilanz. SALCA-Schwermetall.

IPCC, 2006. Volume 4: Agriculture, Forestry and Other Land Use, IPCC guidelines for national greenhouse gas inventories.

Keller, T., Desaules A., 2001. Böden der Schweiz: Schadstoffgehalte und Orientierungs-werte (1990 – 1996).

Koch, P., Salou, T., 2013. AGRIBALYSE ® : Methodology - Version 1.1.

Nemecek, T., Schnetzer, J., 2011. Methods of assessment of direct field emissions for LCIs of agricultural production systems - Data v3.0.

Oberholzer, B., Poser, K., Dill, A., Herzog, F., 2001. Kann die Nitratauswaschung zuverlässig simuliert werden? In: Neue Erkenntnisse zu Stickstoffflüssen im Ackerbau.

Prasuhn, V., 2006. Erfassung der PO 4 -Austräge für die Ökobilanzierung. SALCA-Phosphor.

Richner, W., Oberholzer, H.R., Knuchel, F., R. O., H., Ott, S., Nemecek, T., Walther, U., 2014. Modell zur Beurteilung der Nitratauswaschung in Ökobilanzen - SALCA-NO3.

RMQS, 2013. Données d’Analyses de Terres.

USDA – Agricultural Research Service, 2005. Revised Universal Soil Loss Equation – Version 2 (RUSLE2). Washington D.C.
